# Attempt to Quantify Molecules of Host Plant Volatiles Evoking an Electroantennographic Response in *Anoplophora glabripennis* Antennae

**DOI:** 10.3390/insects16080781

**Published:** 2025-07-30

**Authors:** Rui Zhang, Jian-Ming Shi, Yi-Bei Jiang, Hui-Quan Sun, Dan-Dan Cao, Hui-Ling Hao, Jian-Rong Wei

**Affiliations:** 1 School of Life Sciences, Hebei Basic Science Center for Biotic Interaction, Hebei University, Baoding 071002, China; zzhang2022132@163.com (R.Z.); 18434072142@163.com (J.-M.S.); jiangyibei123@163.com (Y.-B.J.); hhsunhq@163.com (H.-Q.S.); 2 Research Center of Biotechnology, Hebei University, Baoding 071002, China; caodandan666@163.com; 3 Naval Medical Center, People’s Liberation Army of China, Shanghai 200433, China

**Keywords:** Asian longhorn beetle, host volatiles, electroantennogram, antenna potential

## Abstract

*Anoplophora glabripennis*, is one of the most devastating wood borers of many broad leaf trees. Our previous results indicated that antennae of *A. glabripennis* showed electroantennogram (EAG) responses to several host plant volatiles. However, the quantities of active compounds necessary to trigger an EAG response remains unclear. To relate EAG responses with quantities of active molecules, we quantified the level of molecular triggering in the EAG response of *A. glabripennis* by a series of procedures. First, we used the EAG apparatus to measure EAG responses of *A. glabripennis* to five concentrations of eight chemicals and obtained dose–response curves. Second, volatiles released after blowing air over filter paper loaded with volatiles for different numbers of times (purging) were collected by solid-phase microextraction (SPME) and quantified by gas chromatography (GC), so we obtained the quantity of chemical released from each purge; the minimum number of molecules in each purge in the EAG was calculated by the molar mass for different compounds. For instance, the number of molecules of (*Z*)-3-hexenol reaching the female antennal segment in EAG was 8.68 × 10^8^ at 0.01 ng/μL concentration, and 1.39 × 10^5^ at 0.01 mV potential value. Finally, by comparing sensilla numbers on tested antennal segments with the entire antennae, the minimum number of molecules, or molecular flow, of tested compounds required to elicit an electrophysiological response from two antennae of ALB could be estimated either at a minimum concentration (2.49 × 10^8^ at 0.01 ng/μL concentration of (*Z*)-3-Hexenol, for female) or at a minimum potentiometric response value (3.99 × 10^4^ at 0.01 mV potential value).

## Simple Summary

*Anoplophora glabripennis* is a serious wood borer and quarantine pest in the world. We first studied the minimum number of molecules of several active compounds necessary to trigger an EAG response of *A. glabripennis*. Then by a series of experiments and calculations, the minimum number of molecules, or molecular flow, of tested compounds required to elicit an electrophysiological response from two antennae of *A. glabripennis* were estimated, either at a minimum concentration or at a minimum potentiometric response value, which will help to decide whether *A. glabripennis* would respond to a certain number of molecules in the air in the field.

## 1. Introduction

The electroantennogram (EAG) is an electrophysiological technique commonly used to study the olfactory responses of insects [1]. By measuring the electrical signals from the antennae of insects, it is possible to visually detect responses to chemical signals. The EAG can be used for the following: screening biologically active substances; identifying active chromatographic components; selecting active synthetic compounds; field monitoring of odor concentrations; and as a bio-detector in gas spectrometry [2,3,4]. An EAG can provide accurate data on the activity of the insect olfactory system and has been widely used in the study of chemical ecology [5]. EAG values can provide valuable information about an insect’s perception of a particular chemical, but it does not closely relate to the concentration of a chemical (dose) required in a trap to capture an insect in the field; selection of trapping dose is influenced by many factors [6] but amongst them, olfactory response is the primary requirement. As an olfactory detector, the Minimum Detection Limit (MDL) of EAG refers to the lowest concentration of a chemical that can be reliably detected in an EAG system. The MDL of the EAG is usually influenced by the sensitivity of the detection system, and the type of compound tested. Until now, we have not found any research on the quantification of the number of molecules necessary to reach the antenna and trigger a response in an EAG experiment [7].

*Anoplophora glabripennis* (Motschulsky) (Coleoptera: Cerambycidae) is a forest pest native to East Asia [8]. Its preferred host trees mainly belong to the genera *Populus*, *Acer*, *Salix* and *Ulmus* [9,10,11,12]. Adult *A. glabripennis* lay their eggs under the bark of the tree trunk and larvae burrow into the trunk and branches, resulting in the weakening or death of the trees [13]. Outbreak and damage caused by *A. glabripennis* has caused serious consequences in China, some Europe countries and North America, resulting in huge economic losses and ecological destruction of local ecosystems and urban landscapes, and it has been listed as an important quarantine pest in many countries [8,14,15,16].

Volatiles play a crucial role in the complex system of insect–plant interactions. Volatiles released by plants constitute important chemical cues for insects to perceive their environment [17]. Numerous studies have clearly demonstrated that these volatiles influence a wide range of insect behaviors, including host location, oviposition choice and foraging activity [18,19]. Our previous results showed that antennae of *A. glabripennis* had obvious electrophysiological (EAG) responses to the volatiles (*E*)-2-hexenal, hexyl acetate, (*Z*)-3-hexenol acetate, 1-hexanol, (*Z*)-3-hexenol, β-caryophyllene, salicylaldehyde [20] and 3-carene [21]. As a longhorn beetle, the long antennae of *A. glabripennis* adults are constantly waving to detect odors in the air for mating and feeding purposes. However, the number of molecules of these active compounds that are needed to trigger an antennal response is not known.

In this paper we estimated the minimum number of molecules necessary to trigger EAG responses in *A. glabripennis* using a series of procedures:

① We established standard curves of dose–peak area of eight volatiles under constant GC conditions; ② we used the EAG apparatus to determine the response of *A. glabripennis* to five concentrations of volatiles to obtain dose–response curves and estimate electrical potential values at particular doses or the dose at selected electrical potential values; ③ we collected volatiles released from loaded filter paper using solid-phase microextraction-gas chromatography (SPME) coupled with GC so the quantity of volatiles and number of molecules released could be calculated; ④ we measured the ratio of the surface area of tested antennal flagellum to the cross-sectional area of the EAG glass nozzle setting to calculate the number of molecules reaching the tested flagellum; ⑤ we estimated the minimum number of molecules on the tested flagellum at a certain EAG value or at a certain concentration; ⑥ we quantified the number of sensilla on the entire *A. glabripennis* antenna; ⑦ thus, the minimum number of molecules for each compound (at certain time, 0.5 s for EAG setting) required to stimulate the entire antenna could be estimated for further evaluation.

This research helps establish the relationship between the quantity of volatiles to which insects are exposed in their natural environment and their ability to receive and use these molecules as signals. For *A. glabripennis*, the objective of this study was to quantify the minimum number of volatile molecules needed to trigger the antennal response in EAG and estimated the required molecule quantities for the entire antennae of an individual adult.

## 2. Material and Methods

### 2.1. Material

Insects: *A. glabripennis* adults were collected from infested trunks of *Salix babylonica* L. in Baoding city, Hebei Province, China in early-May 2024. After tree felling in early spring, log segments (diameter: 30–120 cm, length: 30–50 cm) were transported back to the laboratory. Cut sections of logs were sealed with paraffin wax and the logs were held in steel mesh-covered cages (23 ± 2 °C) until adults emerged. Adults were collected and kept individually in clear plastic containers (18 cm × 11 cm × 8 cm, PE) and fed regularly every two days with twigs and leaves of *Elaeagnus angustifolia* (1 cm diameter, 5 cm length), at 25 ± 2 °C, 70 ± 5% relative humidity (RH), 14 h:10 h (light/dark) and ventilation at regular intervals to keep the environment clean.

Volatiles: The eight plant volatiles used in the experiment were (*Z*)-3-hexenol (98%, J&K Co., Ltd., Gyeonggi, Republic of Korea), (*Z*)-3-hexenol acetate (≥98%, Aldrich Co., Ltd., London, UK), (*E*)-2-hexenal (98%, Aldrich), 1-hexanol (98%, Fluka, Geneva, Switzerland), β-caryophyllene (98.5%, Fluka), salicylaldehyde (99%, J&K), hexyl acetate (>99%, TCI Co., Ltd., Taipei, Taiwan) and 3-carene (90%, J&K).

Volatile presentation: In the EAG test and subsequent odor release tests, volatiles were presented in Pasteur pipettes (7 mm × 230 mm) on filter paper strips as the chemical carrier, filter paper (Hangzhou Jiayang North Pulp Paper Co., Ltd., Hangzhou, China), was cut into 2.5 cm × 1.2 cm strips and folded to 2.5 cm × 0.6 cm. The size of the paper strip was the same throughout all experiments. A stimulated gas flow controller Syntech CS-55 (Kirchzarten, Germany) was used to administer the volatiles.

Volatile collection: To effectively collect trace amounts of volatiles, a manual solid-phase microextraction (SPME) (Supelco Co., Ltd., Bellefonte, PA, USA) with gray extraction head of DVB/CAR/PDMS (divinylbenzene/carboxyethyl/polydimethylsiloxane, 50/30 μm partially crosslinked) was used.

### 2.2. Methods

#### 2.2.1. Dose Response of EAG to Different Volatiles

The EAG system consisted of an intelligent data acquisition controller IDAC-2, a micro-manipulator Syntech MN-151, a stimulus airflow controller Syntech CS-55 and the Syntech 1.2.5 software processing system (Syntech Co., Ltd., Steinfeld, Germany). The odor delivery system and stimulation method were similar to the experimental design described by Yan et al. [22].

Active adult females and males were selected and a razor blade used to cut off the first two segments from the tip of the antennae, and then the top antennal segment removed quickly. The second flagellum from the tip is the most sensitive part of the antenna and was used in the EAG [23], and the flagellum were connected with glass electrode with saline solution. Each sample solution (20 µL) was placed onto a filter paper strip and two minutes later placed into a Pasteur pipette, the tip of which was connected to the gas stimulus control device. The gas flow rate was set to 400 mL/min. The stimulation duration was 0.5 s, with a minimum interval of 1 min between consecutive stimuli. The voltage measurement was set to 1 mV. When measuring the EAG responses of *A. glabripennis* adults to single compounds at different concentrations, n-hexane was used as the control. Twenty microliters of five concentrations of sample solution (1 ng/μL, 10 ng/μL, 100 ng/μL, 1 μg/μL, 10 μg/μL) were tested, respectively, in order of increasing concentrations, as well as the control (0 ng/μL [n-hexane only]). Every concentration of tested compound was evaluated three times on one antenna. Because the activity of antennae decreases over time, the activity of antennae must be calibrated: 2 mol/L (*Z*)-3-hexenol samples were used before and after EAG determination to eliminate the influence of decreasing antennal activity and differences between individual antennae.

#### 2.2.2. Quantifying Volatile Compounds Released from Filter Paper Strips in a Single Purge

For each purchased compound (for convenience, we call them pure compounds in the subsequent text) 1 μL was added to filter paper strips, specifically, (*E*)-2-hexenal, hexyl acetate, (*Z*)-3-hexenol acetate, 1-hexanol, (*Z*)-3-hexenol, β-caryophyllene, salicylaldehyde or 3-carene. The treated filter paper strips were then purged (single blowing over the loaded filter paper) in a pulsed airflow of 400 mL/min and the volatile released from the filter paper collected by a manual SPME with the extraction head (to absorb the majority of molecules) inserted into the Pasteur tube. Each compound was pulse-purged 5, 7, 9, 11 and 13 times, respectively, and then the SPME was immediately injected into a gas chromatograph (GC) to detect the peak areas. Gas chromatographic conditions were as follows: HP-5 column (30 m × 320 µm × 0.25 µm); heating procedure was as follows: starting from 40 °C, held for 1 min; increased to 180 °C at the rate of 8 °C/min, held for 1 min; then increased to 200 °C at the rate of 20 °C/min, held for 2 min. The detector was at 250 °C. The sample was injected without splitting, and the temperature of the injection port was 240 °C; the carrier gas was nitrogen. The chromatographic peaks were integrated after injection and the peak areas were recorded. Therefore, the mean peak area of each purge and each chemical could be accurately calculated.

To simplify analysis, we assumed that a release pattern of 20 μL of the chemical solution on the filter paper was similar to that of 1 μL of pure chemical (≈1 mg). Thus, another 20 μL (50 μg/μL) of the compound (≈1 mg) was added onto a strip of filter paper, purged three times, absorbed by SPME and immediately injected into the GC. Each extraction treatment was repeated three times.

With the same GC conditions and procedures, standard curves of for each tested chemical were established at each concentration: 0.5, 2.5, 5, 25 and 50 μg/μL. From this standard curve, the average amount of volatile chemical in one purge of 1 μL of pure chemical was calculated. Moreover, each purge of different dose (concentration) of compound could be calculated.

#### 2.2.3. The Ratio of Chemical Contact Surface Area of the Tested Antennal Segment to Cross-Sectional Area of Odor Delivery in a Single Purge

The odor delivery system and stimulation method were as described by Yan et al. (2005) [22]: the continuous air stream was filtered through activated carbon, humidified with distilled water and then blown at 100 mL/min through a glass nozzle with a diameter of 0.6 cm to the antenna. The distance from the outlet of the nozzle to the antenna was approximately 1 cm. A three-way valve was provided in the air circuit so that when the valve for the air flowing through the tested compound was closed, the clean air flowed continuously over the antenna; when the valve for the air flowing through the tested compound was open (0.5 s), part of the airflow passed through a Pasteur pipette (outlet diameter 2 mm) and within it, a strip of filter paper containing the volatile compound under evaluation, and the mixed airflow passed through the glass nozzle and over the antenna. The surface area of the tested antennal segment was calculated, and the cross-sectional area of the glass nozzle was also calculated. By comparing both areas as a ratio, the amount of compound contacting the surface of the tested antennal segment during each purge could be further estimated. The formula for calculation of the ratio was as follows:

① Calculation of the surface area of the second segment from the tip of the antenna. Assuming that the total surface area of the second segment of the antenna is A_1_ (in square millimeters), r_1_ is the radius of the antenna (in millimeters) and l is the length of the antenna segment (in millimeters). The shape of the *A. glabripennis* antennal could be treated as cylindrical, so the antenna surface area could be calculated: A_1_ = 2πr_1_l.

② Calculation of the cross-sectional area of the glass nozzle (odor blow out). Assuming that the radius of the glass tube is *r*_2_, and the cross-sectional area of the odor diffusion is *A*_2_, the area can be expressed as *A*_2_ = π*r*_2_^2^.

③ Calculation of the ratio of the antennal segment to the cross-sectional area of the odor diffusion. *R* = *A*_1_/*A*_2._

#### 2.2.4. Quantification of the Number of Sensilla on an *A. glabripennis* Antenna

Antennae were carefully removed from the base of the antennal fossa with forceps under a microscope. After a series of standard scanning electron microscopy (SEM) treatments and observation, the morphological characteristics, types and distribution of different sensilla were recorded under Phenom SEM [24]. The working voltage of the electron microscope was 10.0 kV. The antennal sensilla were identified [25], and the number of sensilla trichodea and sensilla basiconica on antennal segments were counted.

### 2.3. Estimation of the Minimum Number of Molecules Necessary to Trigger the EAG Response in A. glabripennis

From the EAG dose–response equation, we defined a minimum dose and then calculated the minimum potential value for each chemical that was necessary to trigger the antennal response. The number of molecules released by a purge at the minimum dose was calculated using the equation obtained above for the ‘amount of compound released from each filter paper strip in a single purge’ and then the minimum number of molecules reaching the antenna could be calculated from the results for ‘the ratio of chemical contact surface area of the tested antennal segment to odour delivery opening in a single purge’.

The ratio of sensilla number on the tested antennal segment to the total number of sensilla on the entire antenna was used to calculate the minimum number of molecules present (after certain durations) in the air to trigger the response of entire antennae of *A. glabripennis*. This could be calculated by the equation *s/S = T/t*. Where *s* refers to the number of sensilla on the second segment, *S* refers to the number of sensilla on an entire antenna, *T* refers to the number of molecules required to elicit an electrophysiological response in the whole antenna and *t* refers to the number of molecules swept onto (or absorbed by) the tested second segment.

### 2.4. Statistical Analysis

EAG response data used in analyses were the average of the sample EAG measurements minus the control measurements. EAGs of compounds were analyzed by one-way ANOVA followed by Duncan’s method for multiple comparisons to determine variability. Data for different numbers of purges and quantities of compound released were analyzed by descriptive statistical analysis.

The morphology and distribution of antennal sensilla were recorded, and all sensilla were counted for both sexes. The types of antennal sensilla were mainly classified using the nomenclature system of Schneider [26] and Zacharuk [27,28]. Three antennae were investigated for males and for females. Data were statistically analyzed and the results were expressed as mean ± standard error (SE).

Data were analyzed using IBM SPSS Statistics 26.0 software and plotted using Origin (Version: 2022).

## 3. Results

### 3.1. Adult EAG Responses to Eight Volatiles

EAG responses of female and male *A. glabripennis* to eight volatiles at five concentrations of 1 ng/μL, 10 ng/μL, 100 ng/μL, 1 μg/μL and 10 μg/μL were calculated and analyzed. The mean values for EAG responses of female and male ALB to eight volatiles were positively correlated with concentration, and different equations were fitted, as shown in Table 1.

### 3.2. Quantity of Compound Released from Filter Papers

Standard curve equations of the eight compounds are shown in Table 2, where *Y* represents the compound concentration and *X* represents the chromatographic peak area.

The eight compounds released from the filter papers after different numbers of purges were collected by SPME and analyzed by GC. The quantities of released compound showed a linear increasing trend as purge number increased (Table 3).

According to the equations in Table 3, how much of the compound released by one purge was obtained. Based on the molar mass, the number of molecules released by one purge from 1 μL of the pure compound on a filter paper strip was calculated, and the results are shown in Table 4. The mean number of molecules released in one purge from a compound (20 μL, 50 μg/μL) on the filter paper carrier was obtained by purging on the strip three times, and the results are also shown in Table 4.

### 3.3. Estimation of Minimum Values

#### 3.3.1. Calculation of the EAG Value Triggered by 0.01 Ng/μL of Compound

If we set the concentration (*X*) of the equation in Table 1 as 0.01 ng/μL, then the corresponding potential value of the compound perceived by *A. glabripennis* antennae in the EAG experiment could be calculated, as shown in Table 5. This indicated that the sensitivity of antennae of *A. glabripennis* depended on the compound tested, and almost all values were over 0.01 mV.

#### 3.3.2. Calculation of the Compound Dose Corresponding to a 0.01 mV Potential Response Value

If we assume that the minimum potential value that can be perceived by *A. glabripennis* antennae in an EAG experiment is 0.01 mV, according to the equation in Table 1, then the corresponding concentrations of different compounds can be calculated, as seen in Table 6.

### 3.4. Surface Area of the Tested Antennal Segment in EAG Setup

We measured the diameter and length of the second segment (from tip) of five male and five female antennae. The second segment of male antennae had mean diameters of 0.5 ± 0.02 mm (standard error, SE), length of 4 ± 0.16 mm (SE) and surface area of 6.28 ± 0.45 mm^2^. The second segment of female antennae had mean diameters of 0.48 ± 0.02 mm (standard error, SE), length of 2.96 ± 0.11 mm (SE) and surface area of 4.46 ± 0.22 mm^2^.

### 3.5. Number of Molecules Reaching the Tested Antennal Segment at a Concentration of 0.01 Ng/μL or Stimulating the Potential Value of 0.01 mV

Results showed that the radius of the glass nozzle (cross-section of odor diffusion opening) was 3 mm so that the cross-section area of odor diffusion was approximately 28.26 mm^2^. According to this result and the surface area of tested antennal segments, the ratio of the cross-section of the odor diffusion to the male antenna surface was calculated as 4.50:1 and to the surface area of the female antenna as 6.34:1.

If we set the chemical concentration at 0.01 ng/μL (adding 20 μL on the filter paper strip), the numbers of molecules of tested chemicals reaching the antennal segment in EAG could be calculated and seen in Table 7. If we set the potential value as 0.01 mV or at the potential value of 0.01 mV, the number of molecules of tested chemicals reaching the antennal segment in EAG are also shown in Table 7.

### 3.6. Number of Antennal Sensilla

Observations of adult antennae by SEM revealed that the two main types of sensilla that served as chemoreceptors were distributed on the antennae of *A. glabripennis* adults: trichodea sensilla (*t*) and basiconic sensilla (*b*). We took photos of the entire female and male antennae, as well as their eighth flagellomere (Supplement Figure S3). The number of these two types of sensilla are shown in Table 8. The total number of trichodea sensilla and basiconic sensilla on the second segment of the male antenna (flagellum 8) was 502 ± 22. The total number of trichodea sensilla and basiconic sensilla on the second segment of the female antenna (flagellum 8) was 467 ± 27.

Calculations showed that the ratio of the number of sensilla on the second segment of male antennae to the number of sensilla on the entire antenna was 502/3416 ≈ 14.70%. The ratio of the number of sensilla on the second segment of female antennae to the number of sensilla on the entire antenna was 467/3252 ≈ 14.36%.

### 3.7. Number of Molecules Required to Trigger an Electrophysiological Response in the Entire Antenna

If the number of molecules necessary to trigger a response in the tested antennal segment is equal to that of the whole insect antenna, then the number of molecules flowing past the whole antenna over a certain period could be reduced, since the total number of sensilla on the entire antenna is larger than that on the second antennal segment alone, if all sensilla are triggered. Therefore, the number of molecules required to elicit an electrophysiological response in both the entire antennae (two) at the concentration of 0.01 ng/μL could be calculated and is shown in Table 9. At a potentiometric response value of 0.01 mV, the number of molecules causing the reaction is also calculated and shown in Table 9.

## 4. Discussion

Antennae are the main sensory organs of insects, and insect chemoreceptors, are largely distributed on the antennae, and play a key role in insect behavior including courtship, host recognition and localization [29]. Most olfactory receptors distributed on the antennae enable detection of chemicals with high sensitivity and selectivity. Multiple morphological and physiological types of olfactory receptors are present on insect antennae, each of which containing one or more olfactory receptor neurons (ORNs) involved in olfactory perception. The response of ORNs to these chemical signals can be monitored by electrophysiological techniques such as EAG and single-sensilla recording (SSR) [30,31].

In our experiments, we evaluated EAG responses of ALB to eight host plant volatiles at five concentrations. Thresholds relating to the quantity of chemicals present are physiologically important in insect perception [32], and thresholds for chemical doses were calculated by fitting a dose–response curve and equations. Thresholds of chemical doses may be related to the chemical nature of the compound, volatility and sensitivity of the receptors. At the same time, the value that can be detected by the EAG may be much higher than the actual level perceived by insect antennae in nature [33]. When air carries odor molecules and forms irregular eddy diffusion patterns, insects perceive these odors through their olfactory organs and thus communicate or respond behaviorally [34]. When calculating the contact area of the tested antennae, we used the full surface area of the antennal segment, because when the chemical was blown towards the antenna, the gas flow produced complex aerodynamics on the surface, including formation of vortex phenomena. Therefore, the contact area was an estimated value.

Plant volatiles play a crucial role in orienting insects to host plants by releasing specific chemicals that send signals directing insects to suitable plants for breeding or foraging [35,36]. Filter paper strips are commonly used carriers in EAG studies. In this study, by purging air over the filter, we could release known quantities of eight volatiles in a single purge. In this blowing adsorption experiment, we first used Tenax adsorbent to trap volatiles, but it turned out to be difficult to adsorb the trace volatiles to perform quantity analysis in GC, so we used solid-phase microextraction adsorption techniques. Certainly, there are drawbacks to using SPME to conduct chemical quantification, but in our experimental set, the very thin output of a Pasteur pipette can guarantee most of volatiles were absorbed by the SPME. Based on the quantification result, the number of molecules released in a single purge was obtained. Our results on the number of molecules reaching the antennal surface are convincing, because the surface of ALB antennae are rough and densely packed with a large number of densely distributed sensilla that amplify the effective contact area. When volatile molecules diffuse onto the antennal surface, the sensilla act as molecular capture sites, efficiently trapping passing molecules and increasing their attachment rate. Our results provide an important reference for further research on detecting the volatile limits in natural environments [37].

In the EAG experiment, 20 µL of different concentrations of the chemical solution were added dropwise to the filter paper strip to form a circle with an area of approximately 2.54 cm^2^. To determine the number of molecules released in one purge from the loaded filter paper strip, 1 µL of the pure compound (about 1000 μg) was added dropwise to the strip in a circular area of 0.38 cm^2^, so the volume of the solution affected the diffusion area. Usually, a larger diffusion area releases more molecules of the compound in a single purge. To decrease release rate errors from different diffusion areas between 1 μL and 20 μL, we calculated the amount released from 20 μL of 50 μg/μL (about 1000 μg) of tested chemicals (Table 4). Since the data in two columns were not very different, we could logically calculate the release quantity of the minimum concentrations at a volume of 20 μL.

When the parameter *K* in the fitted equation is small and the intercept parameter *b* is large, the initial release of the volatiles from filter paper strips is relatively large according to the model (Table 3), for example, (*E*)-2-hexenal. This indicated that the first purge of air can release a large quantity of the chemical on filter paper strips at the initial stage of the experiment, and then the amount of chemical collected increased at a relatively stable rate as the purges continued. The method used to determine the release rate of compound from the filter paper here could be used to decide the lure release rate from the carrier.

Minimum potential detection values calculated (Table 5) provide a reference for improving detection sensitivity of future EAG apparatuses. In order to detect low doses of active chemicals, future EAG settings need to be more sensitive, including the use of high sensitivity amplifiers or improved electrode materials, which may help to capture small fluctuations. At the same time, noise suppression capability is also crucial; the use of higher quality shielding materials and improved signal processing algorithms may improve the signal-to-noise ratio.

The number of molecules required to trigger the entire antennae to respond (Table 9) were less for (*Z*)-3-hexenol, (*Z*)-3-hexenyl acetate and β-caryophyllene compared with other compounds, suggesting that these three compounds were more efficient in activating the antennal response. However, their specific biological function and relative effectiveness needs to be further investigated for use in the behavioral control of *A. glabripennis*.

Though we obtained the minimum number of molecules which could arouse the EAG response of *A. glabripennis*, more research is still required to connect the volatiles in the air released from the host tree or pheromones from other individual beetles (males produced pheromones, females produced short range attractants from oxidizing products on their cuticle, females produced trail pheromones and short range contact pheromones) with the antennal response [8,12,16,38,39,40,41,42]. In fact, our next study will focus on how many molecules can be detected on an aeration cartridge at a certain distance from the host tree. This work should firstly be performed in a fume hood before field testing. We have already finished the field experiment on how far away *A. glabripennis* adults can be while still responding and flying to the target host tree *Populus deltoides* ‘Shalinyang’, which could attract the *A. glabripennis* adults but reduced their offspring [43,44].

## 5. Conclusions

The minimum quantities of molecules, or molecular flow, evoking EAG responses in *A. glabripennis* antennae were estimated for eight host plant volatiles. These results are a prerequisite for understanding response initiation in *A. glabripennis* adults when searching for their preferred host trees in the field. If we can further collect and quantify the molecules of these compounds in the air, we can determine how far away the *A. glabripennis* adults can be while still responding to those host tree-released compounds in the field.

## Figures and Tables

**Table 1:** Equations of compounds and EAG response.

Chemicals	Female	Male
(*Z*)-3-Hexenol	*Y* = 0.266 × *X*^0.162^	*Y* = 0.190 × *X*^0.169^
(*Z*)-3-Hexenyl Acetate	*Y* = 0.256 × *X*^0.189^	*Y* = 0.225 × *X*^0.137^
(*E*)-2-Hexenal	*Y* = 0.417 × *X*^0.263^	*Y* = 0.184 × *X*^0.398^
1-Hexanol	*Y* = 0.365 × *X*^0.282^	*Y* = 0.214 × *X*^0.409^
β-caryophyllene	*Y* = 0.176 × *X*^0.136^	*Y* = 0.117 × *X*^0.140^
Salicylaldehyde	*Y* = 0.160 × *X*^0.206^	*Y* = 0.113 × *X*^0.162^
Hexyl acetate	*Y* = 0.300 × *X*^0.210^	*Y* = 0.140 × *X*^0.360^
3-Carene	*Y*= 0.132 × *X*^0.178^	*Y* = 0.117 × *X*^0.190^

Note: *Y* represents EAG response value (mv); *X* represents concentration (μg/μL).

**Table 2:** Standard curve equations for quantitative analysis of eight compounds.

Chemicals	Regression Equation	Correlation Coefficient
(*E*)-2-Hexenal	*Y* = 0.0002*X* + 1.269	0.987
Hexyl acetate	*Y* = 0.0003*X* − 0.194	0.995
(*Z)*-3-Hexenyl Acetate	*Y* = 0.0003*X* + 0.635	0.998
1-Hexanol	*Y* = 0.0002*X* + 0.541	0.983
(*Z*)-3-Hexenol	*Y* = 0.0003*X* + 0.988	0.999
β-caryophyllene	*Y* = 0.0002*X* + 0.090	0.999
Salicylaldehyde	*Y* = 0.0002*X* + 1.648	0.991
3-Carene	*Y* = 0.0002*X* + 0.909	0.999

**Table 3:** Quantities of different purge numbers of pure compound (1 μL) released from a filter paper strip.

Chemicals	Simultaneous Equations
(*Z*)-3-Hexenol	*Y* = 2.290 + 1.502*X*
(*Z*)-3-Hexenyl Acetate	*Y* = −1.947 + 3.684*X*
(*E*)-2-Hexenal	*Y* = 11.043 + 0.964*X*
1-Hexanol	*Y* = 1.467 + 1.196*X*
β-caryophyllene	*Y* = 0.046 × X^1.780^
Salicylaldehyde	*Y* = 0.166 + 1.329*X*
Hexyl acetate	*Y* = −2.112 + 2.986*X*
3-Carene	*Y* = 6.670 + 2.441*X*

Note: *Y* represents the mass (μg); *X* represents the number of purges.

**Table 4:** Number of molecules released from the filter paper strip in a single purge.

Chemicals	Number of Molecules (1 μL Pure Compound)	Number of Molecules (20 μL, 50 μg/μL)
(*Z*)-3-Hexenol	2.28 × 10^16^	2.75 × 10^16^
(*Z*)-3-Hexenyl Acetate	7.35 × 10^15^	1.57 × 10^16^
(*E*)-2-Hexenal	7.36 × 10^16^	3.33 × 10^16^
1-Hexanol	1.57 × 10^16^	1.92 × 10^16^
β-caryophyllene	1.35 × 10^14^	8.33 × 10^14^
Salicylaldehyde	7.37 × 10^15^	7.70 × 10^15^
Hexyl acetate	3.65 × 10^15^	1.22 × 10^16^
3-Carene	4.03 × 10^16^	2.21 × 10^16^

**Table 5:** Electrical potential values (mV) triggered by active compounds at a concentration of 0.01 ng/μL in antennae of *Anoplophora glabripennis* adults.

Compounds	Female	Male
(Z)-3-hexenol	0.041	0.027
(*Z*)-3-hexenol acetate	0.029	0.046
(*E*)-2-hexenal	0.020	0.002
1-hexanol	0.014	0.002
β-caryophyllene	0.037	0.023
salicylaldehyde	0.015	0.018
hexyl acetate	0.027	0.002
3-carene	0.017	0.013

**Table 6:** Concentrations of different chemicals that trigger 0.01 mV EAG in *Anoplophora glabripennis* adults.

Chemicals	Female	Male
(*Z*)-3-Hexenol	1.60 × 10^−9^	2.71 × 10^−8^
(*Z*)-3-Hexenyl Acetate	3.54 × 10^−8^	1.35 × 10^−10^
(*E*)-2-Hexenal	6.91 × 10^−7^	6.64 × 10^−4^
1-Hexanol	2.88 × 10^−6^	5.59 × 10^−4^
β-caryophyllene	6.95 × 10^−10^	2.34 × 10^−8^
Salicylaldehyde	1.43 × 10^−6^	3.16 × 10^−7^
Hexyl acetate	9.25 × 10^−8^	6.55 × 10^−4^
3-Carene	5.07 × 10^−7^	2.50 × 10^−6^

**Table 7:** Number of molecules reaching the antennal segment in the EAG.

Chemicals	0.01 ng/μL Concentration		0.01 mV Potential Value	
	Female	Male	Female	Male
(*Z*)-3-Hexenol	8.68 × 10^8^	1.22 × 10^9^	1.39 × 10^5^	3.31 × 10^6^
(*Z*)-3-Hexenyl Acetate	4.95 × 10^8^	6.98 × 10^8^	1.75 × 10^6^	9.42 × 10^3^
(*E*)-2-Hexenal	1.05 × 10^9^	1.48 × 10^9^	7.26 × 10^7^	9.83 × 10^10^
1-Hexanol	6.06 × 10^8^	8.53 × 10^8^	1.74 × 10^8^	4.77 × 10^10^
β-caryophyllene	2.63 × 10^7^	3.70 × 10^7^	1.83 × 10^3^	8.66 × 10^4^
Salicylaldehyde	2.43 × 10^8^	3.42 × 10^8^	3.47 × 10^7^	1.08 × 10^7^
Hexyl acetate	3.85 × 10^8^	5.42 × 10^8^	3.56 × 10^6^	3.55 × 10^10^
3-Carene	6.97 × 10^8^	9.82 × 10^8^	3.53 × 10^7^	2.46 × 10^8^

**Table 8:** Number (mean ± SE) of sensilla on the antennae of female and male *Anoplophora glabripennis*.

Type of Sensilla	Gender	Scape	Pedicel	Flagellomeres									Total Number
				1	2	3	4	5	6	7	8	9	
*t*	female	0 ± 0	13 ± 0	103 ± 10	111 ± 10	64 ± 3	58 ± 4	52 ± 7	42 ± 1	48 ± 2	43 ± 3	94 ± 8	628 ± 48
*b*	female	0 ± 0	0 ± 0	28 ± 0	288 ± 19	188 ± 20	312 ± 26	427 ± 33	242 ± 14	269 ± 17	424 ± 24	446 ± 30	2624 ± 183
*t*	male	14 ± 0	28 ± 0	164 ± 11	83 ± 2	85 ± 8	67 ± 11	63 ± 8	71 ± 9	61 ± 2	68 ± 6	99 ± 10	803 ± 67
*b*	male	0 ± 0	0 ± 0	27 ± 0	174 ± 16	296 ± 26	225 ± 16	324 ± 29	294 ± 37	450 ± 36	434 ± 16	389 ± 25	2613 ± 201

Note: *t*, mean number of trichodea sensilla; *b*, mean number of basiconic sensilla.

**Table 9:** The minimum number of molecules needed to trigger a response in entire antennae of *A. glabripennis*.

Chemicals	0.01 ng/μL Concentration		0.01 mV Potential Value	
	Female	Male	Female	Male
(*Z*)-3-Hexenol	2.49 × 10^8^	3.59 × 10^8^	3.99 × 10^4^	9.73 × 10^5^
(*Z*)-3-Hexenyl Acetate	1.42 × 10^8^	2.05 × 10^8^	5.03 × 10^5^	2.77 × 10^3^
(*E*)-2-Hexenal	3.02 × 10^8^	4.35 × 10^8^	2.09 × 10^7^	2.89 × 10^10^
1-Hexanol	1.74 × 10^8^	2.51 × 10^8^	5.00 × 10^7^	1.40 × 10^10^
β-caryophyllene	7.55 × 10^6^	1.09 × 10^7^	5.26 × 10^2^	2.55 × 10^4^
Salicylaldehyde	6.98 × 10^7^	1.01 × 10^8^	9.97 × 10^6^	3.18 × 10^6^
Hexyl acetate	1.11 × 10^8^	1.59 × 10^8^	1.02 × 10^6^	1.04 × 10^10^
3-Carene	2.00 × 10^8^	2.89 × 10^8^	1.01 × 10^7^	7.23 × 10^7^

## Data Availability

The original contributions presented in this study are included in the article/Supplementary Materials. Further inquiries can be directed to the corresponding authors.

## Supplementary Materials

The following supporting information can be downloaded at: https://www.mdpi.com/article/10.3390/insects16080781/s1, Supplementary Figure S1: Mean EAG values of *Anoplophora glabripennis* males and females response to eight host volatiles; Supplementary Figure S2: Quantities of chemicals collected from different purge times of pure compound (1 μL) released from filter paper; Supplementary Figure S3: The image of the antennae of male and female *Anoplophora glabripennis*. (a) Male antenna (b) The eighth flagellomere of the male antenna (c) Female antenna (d) The eighth flagellomere of the female antenna; Supplementary Table S1: Compound Information Sheet.
