# Supplementary material for: Attempt to Quantify Molecules of Host Plant Volatiles Evoking an Electroantennographic Response in Anoplophora glabripennis Antennae
Source: Insects. 2025 Jul 30;16(8):781. doi: 10.3390/insects16080781 (PMC12386732; doi:10.3390/insects16080781)
Supplement: Supplementary file 1 [file insects-16-00781-s001.zip › insects-3743518-supplementary.pdf]

Supplement figures

Left: Female **Right**: Male

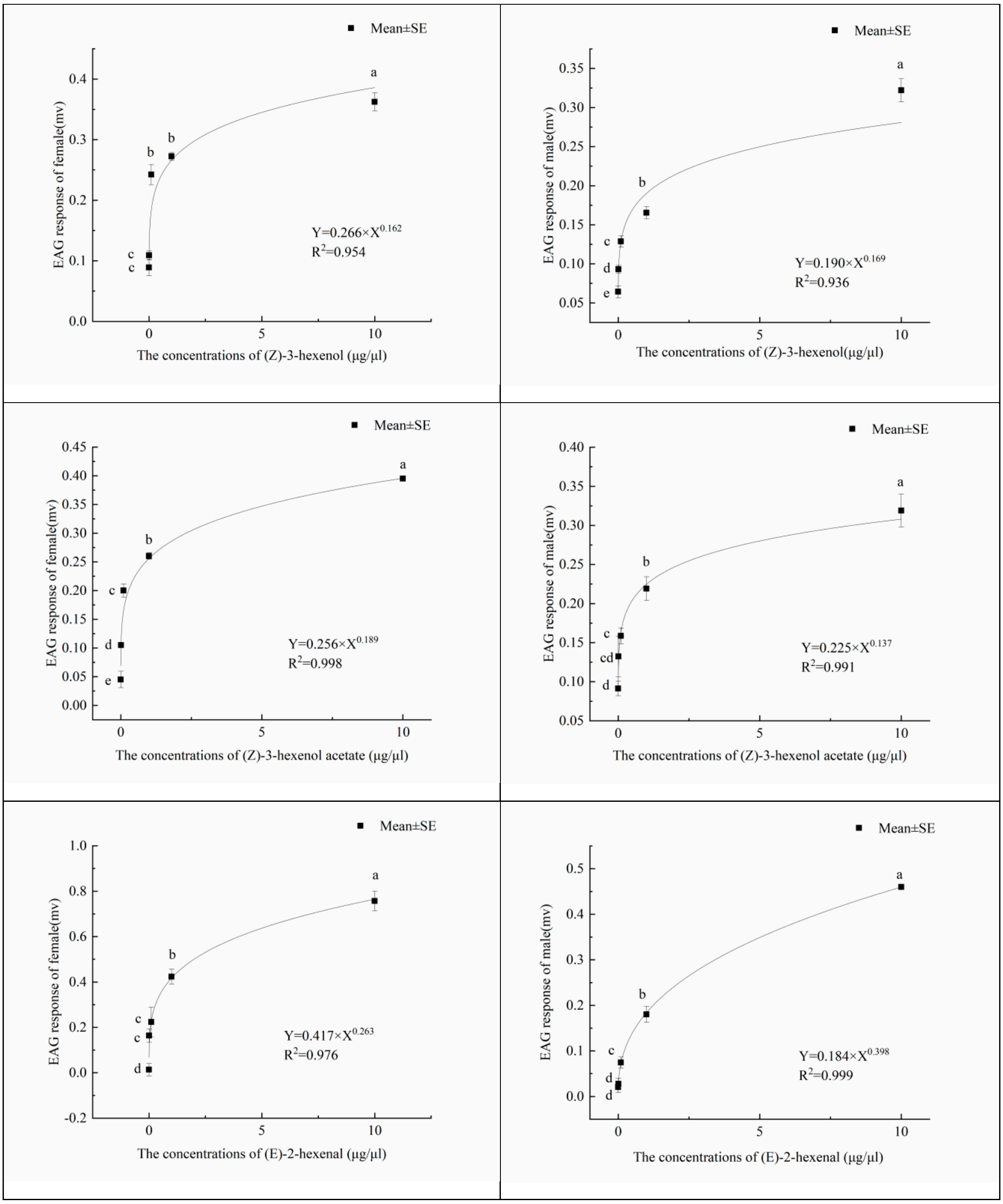

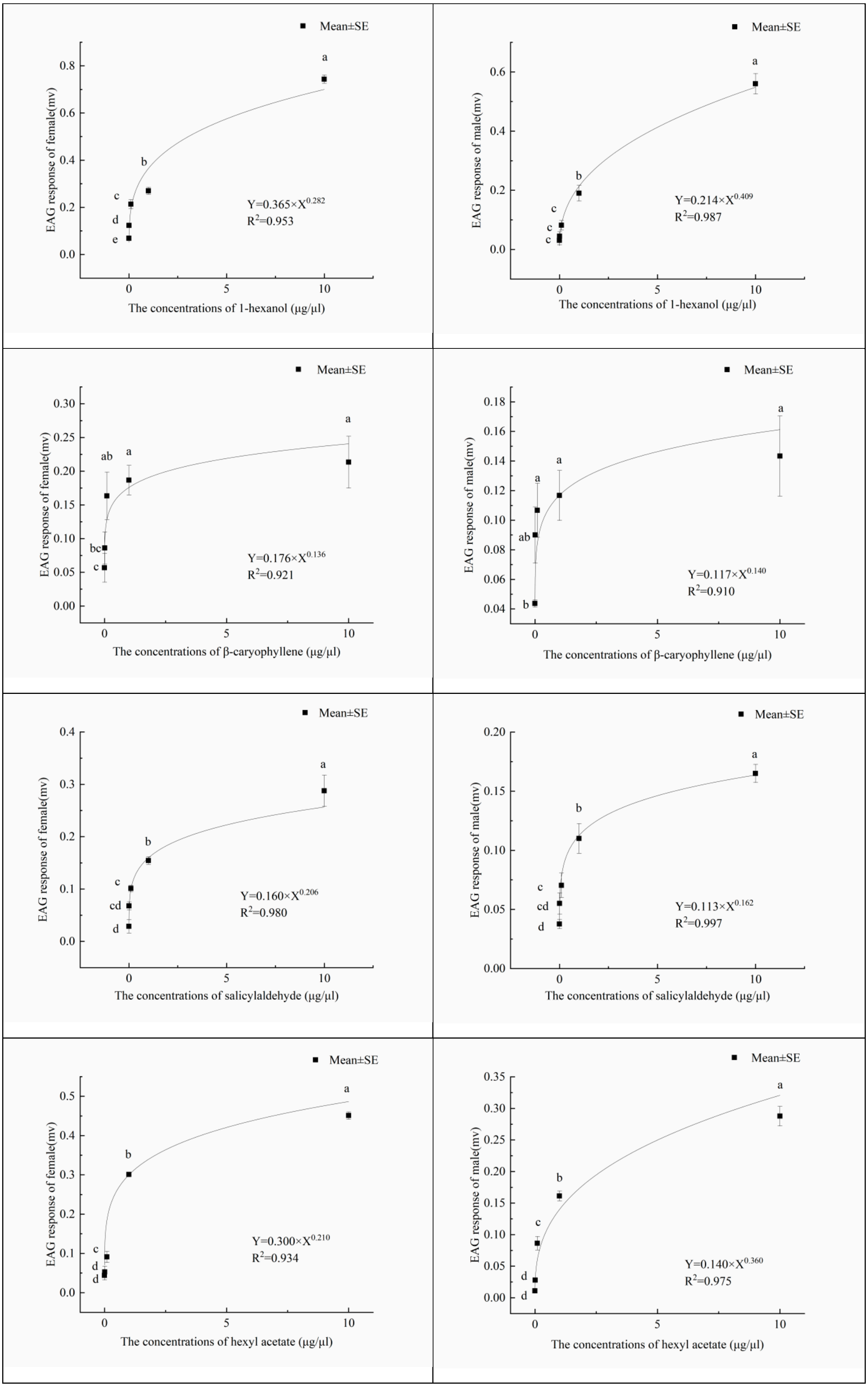

Mean±SE
EAG response of female(mv)
The concentrations of 1-hexanol (μg/μl)
Y=0.365×X^0.282
R^2=0.953
Mean±SE
EAG response of male(mv)
The concentrations of 1-hexanol (μg/μl)
Y=0.214×X^0.409
R^2=0.987
Mean±SE
EAG response of female(mv)
The concentrations of β-caryophyllene (μg/μl)
Y=0.176×X^0.136
R^2=0.921
Mean±SE
EAG response of male(mv)
The concentrations of β-caryophyllene (μg/μl)
Y=0.117×X^0.140
R^2=0.910
Mean±SE
EAG response of female(mv)
The concentrations of salicylaldehyde (μg/μl)
Y=0.160×X^0.206
R^2=0.980
Mean±SE
EAG response of male(mv)
The concentrations of salicylaldehyde (μg/μl)
Y=0.113×X^0.162
R^2=0.997
Mean±SE
EAG response of female(mv)
The concentrations of hexyl acetate (μg/μl)
Y=0.300×X^0.210
R^2=0.934
Mean±SE
EAG response of male(mv)
The concentrations of hexyl acetate (μg/μl)
Y=0.140×X^0.360
R^2=0.975

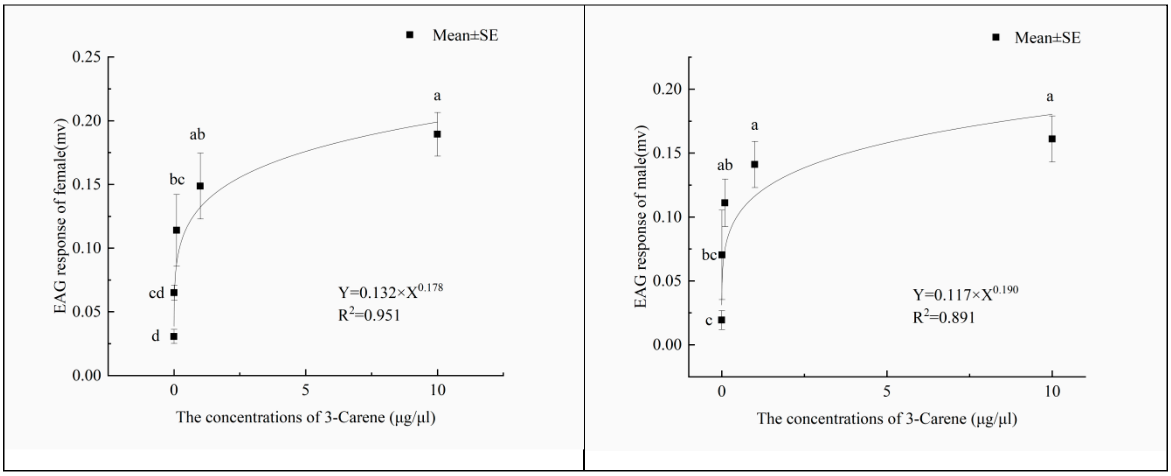

Supplementary Figure S1 Mean EAG values of *Anoplophora glabripennis* males and females response to eight host volatiles

Note: The EAG value is expressed as the mean ± standard error. Different letters indicate significant differences in the EAG responses of *Anoplophora glabripennis* to different concentrations of the same compound at the 0.05 level. One-way ANOVA, multiple comparisons with Duncan test at α=0.05 level.

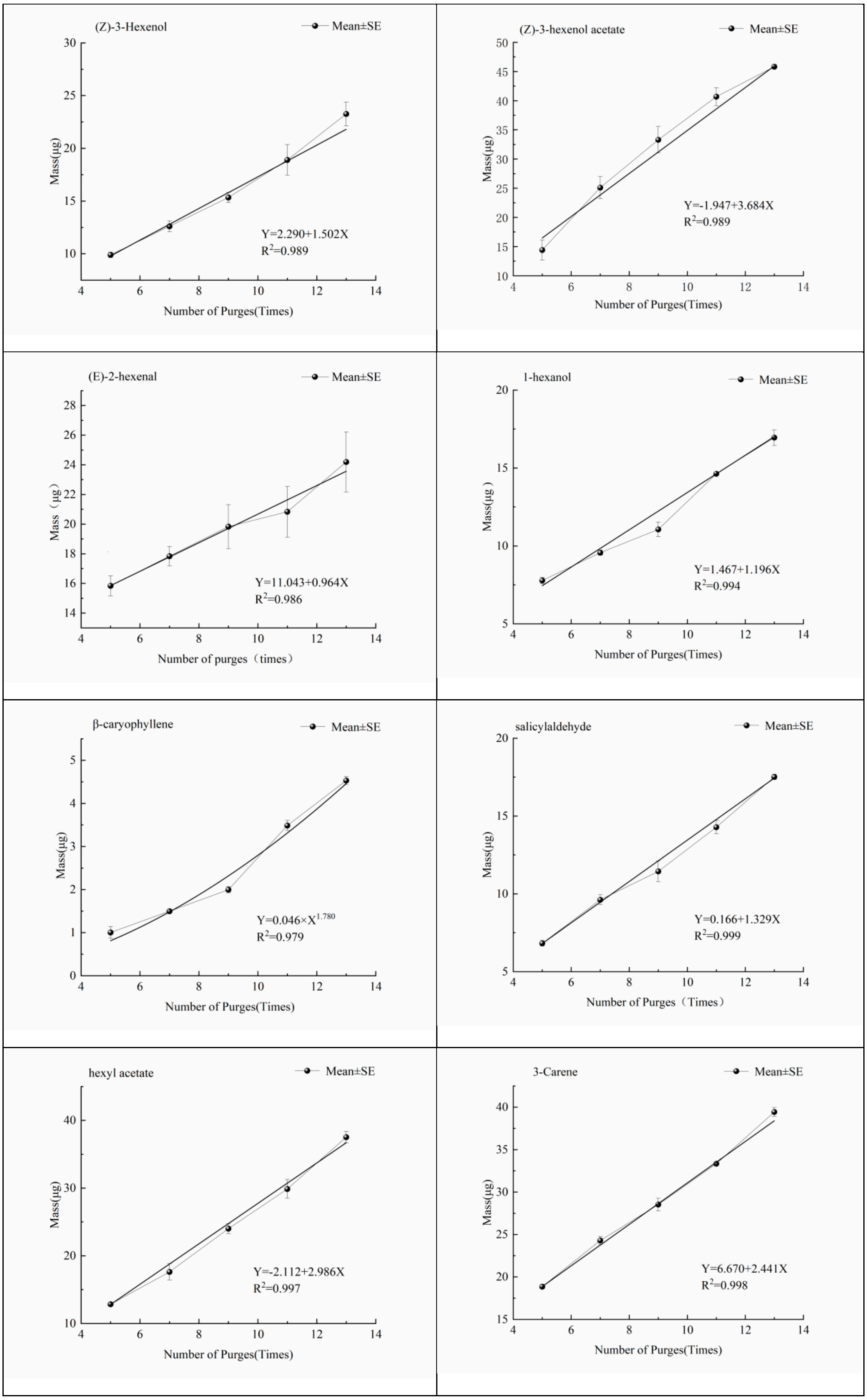

Supplementary Figure S2 Quantities of chemicals collected from different purge times of pure compound (1 μL) released from filter paper

Note: The thin lines represent the average ± standard error of the corresponding quantities at different purge counts, and the thick line represents the fitted linear equation.

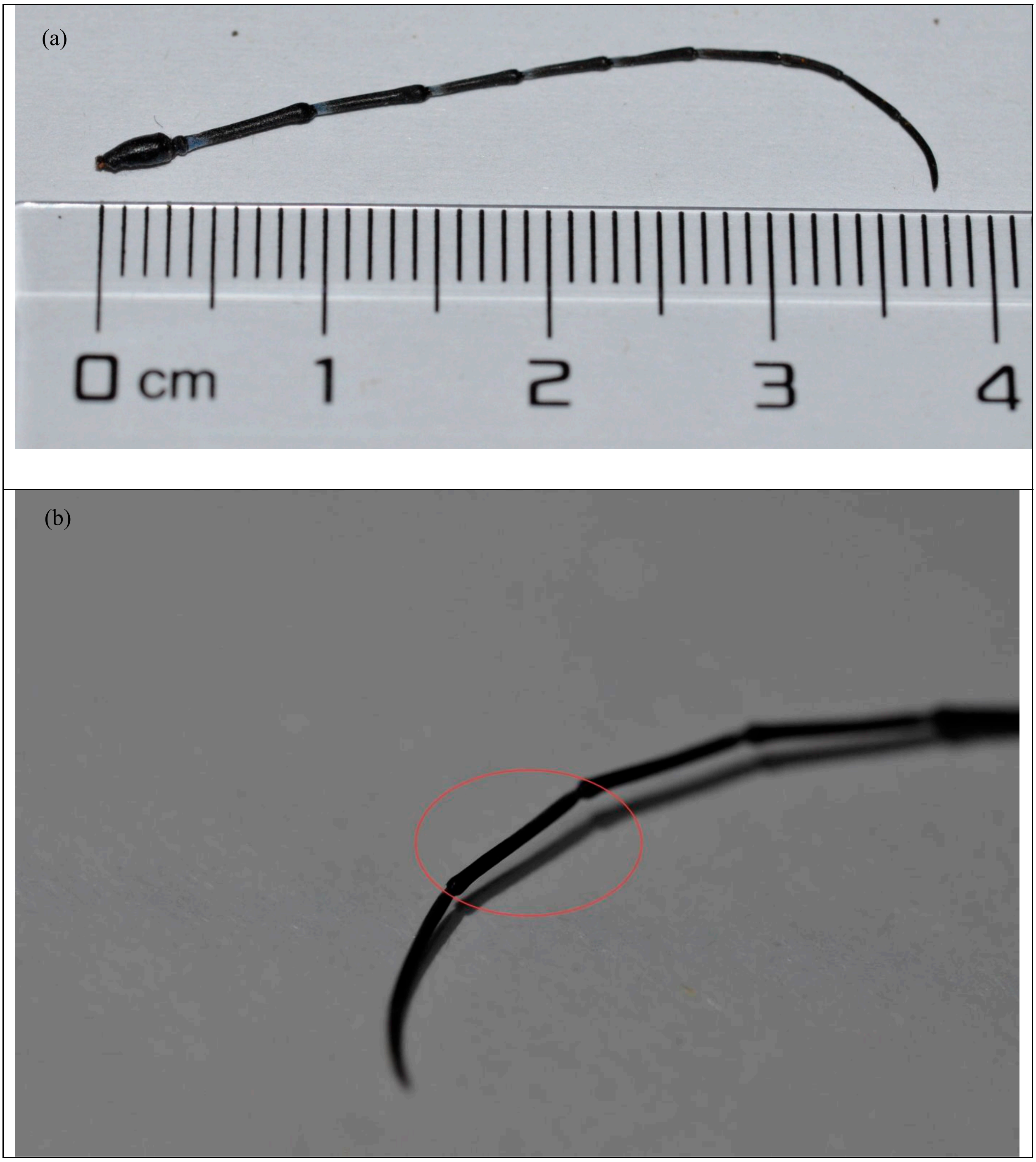
(a)
0 cm 1 2 3 4
(b)

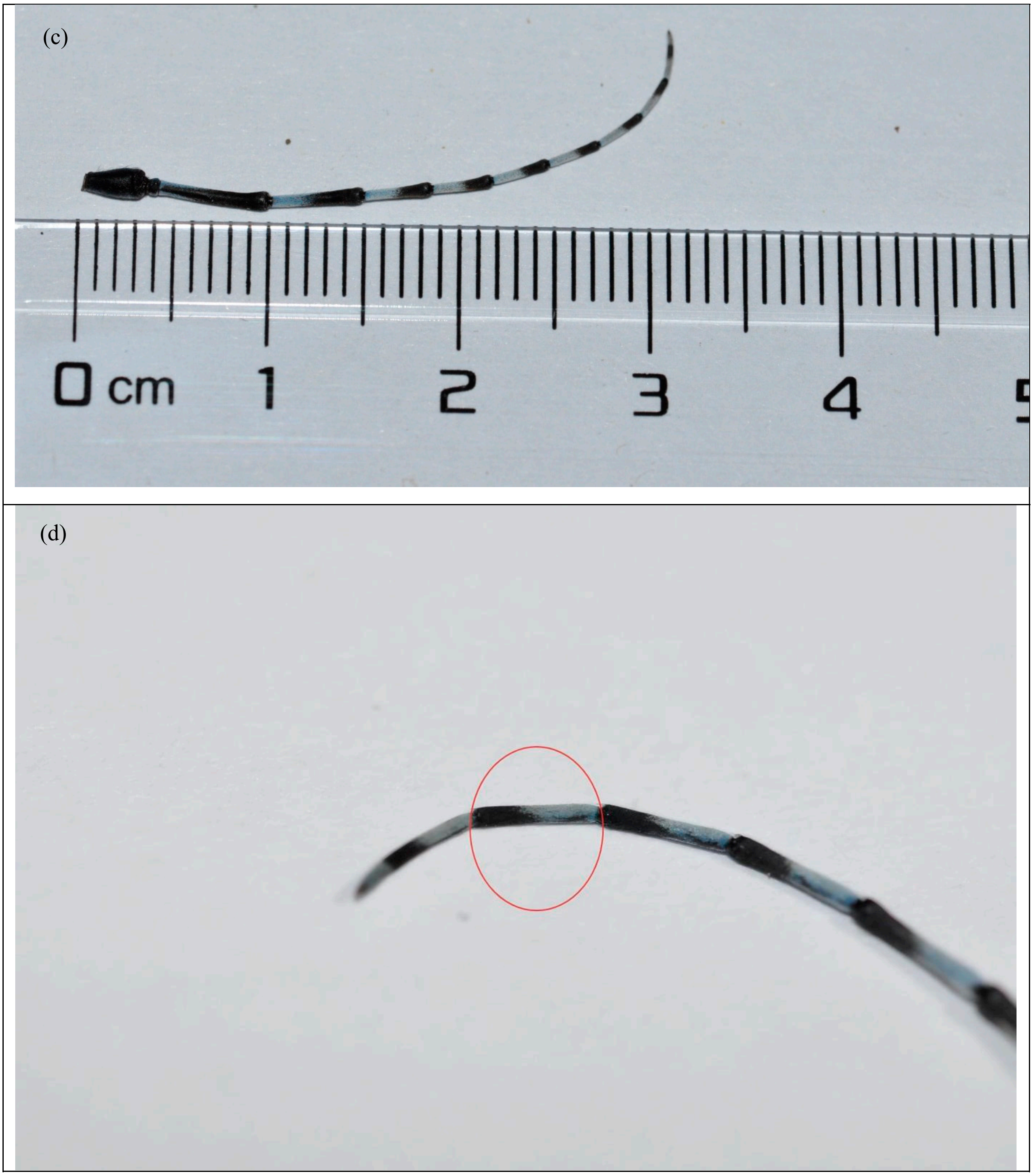

Supplementary Figure S3 The image of the antennae of male and female *Anoplophora glabripennis*

(a)Male antenna (b)The eighth flagellomere of the male antenna (c)Female antenna (d)The eighth flagellomere of the female antenna

Supplementary Table S1 Compound Information Sheet

Chemicals	Molecular formula	Molar mass
(*Z*)-3-Hexenol	$C_6H_{12}O$	100.18
(*Z*)-3-Hexenyl Acetate	$C_8H_{14}O_2$	142.22
(*E*)-2-Hexenal	$C_6H_{10}O$	98.16
1-Hexanol	$C_6H_{14}O$	102.2
β-caryophyllene	$C_{15}H_{24}$	204.39
Salicylaldehyde	$C_7H_6O_2$	122.13
Hexyl acetate	$C_8H_{16}O_2$	144.24
3-Carene	$C_{10}H_{16}$	136.26
